# Serum irisin correlates to the severity of acute myocardial infarction and predicts the postoperative major adverse cardiovascular events

**DOI:** 10.17305/bb.2023.8888

**Published:** 2023-10-01

**Authors:** Qiaoying Chai, Wei Zhang, Lijuan Gao, Yingtao Yang, Shuanli Xin

**Affiliations:** 1 Department of Cardiology, The First Hospital of Handan, Handan, China

**Keywords:** Acute myocardial infarction (AMI), percutaneous coronary intervention (PCI), major adverse cardiovascular events (MACE), irisin, receiver operating characteristic (ROC) curve

## Abstract

Irisin is a myogenic cytokine which plays an important role in the cardiovascular system. The aim of this study was to investigate the correlation between serum irisin levels and major adverse cardiovascular events (MACE) in patients with acute myocardial infarction (AMI) after percutaneous coronary intervention (PCI). A total of 207 patients with AMI who underwent PCI were selected as research subjects. Serum irisin levels at admission were measured, and patients were stratified according to the receiver operating characteristic curve to assess differences in MACE within one year after PCI. After one year of follow-up, 207 patients were divided into two groups, 86 with MACE and 121 without MACE. There were significant differences in age, Killip grade, left ventricular ejection fraction, cardiac troponin I, creatine kinase-muscle/brain, and serum irisin between the two groups. Serum irisin level at admission in AMI patients significantly correlated with the occurrence of MACE after PCI, and could be used as an effective marker for predicting the occurrence of MACE in AMI patients after PCI.

## Introduction

Acute myocardial infarction (AMI) refers to severe myocardial ischemia or necrosis caused by the continuous reduction or termination of blood supply due to acute stenosis or occlusion of coronary arteries [1]. AMI might cause complications, such as chest pain and arrhythmia, with rapid onset and rapid disease progression, which could be life-threatening if not intervened in time [2]. The widespread use of percutaneous coronary intervention (PCI) has significantly reduced mortality in patients with AMI [3]. Nevertheless, studies have shown that prolonged ischemia and ischemia-reperfusion injury might cause myocardial cell apoptosis, which ultimately leads to postoperative major adverse cardiovascular events (MACE) in the short term after AMI and seriously affects the prognosis of patients [4]. Therefore, finding biomarkers to predict the prognosis of AMI is crucial for reducing myocardial infarct size, preventing left ventricular remodeling, reducing the occurrence of MACE, and improving the prognosis of AMI patients [5]. Irisin is a myogenic cytokine discovered in 2012 [6]. Under the regulation of exercise, skeletal muscle cells activate peroxisome proliferator-activated receptor-γ coactivator1α, thereby promoting the expression of fibronectin type III domain-containing protein 5 (FNDC5) [7]. The FNDC5 is processed into a 112 amino acid peptide called irisin [7]. Currently, studies have shown that irisin is mainly released from skeletal muscle, but also expressed in myocardial, liver, lung, neurons, and other tissues [8]. In the study of isolated myocardial ischemia-reperfusion in mice, it was found that irisin could significantly increase the rate pressure product, reduce left ventricular end diastolic pressure, and improve ventricular function, thereby exerting myocardial protection against myocardial ischemia-reperfusion injury [9]. Mechanically, it has been reported that irisin can alleviate cardiac injury through mechanisms, such as mTOR/AMPK/ULK1, autophagy induction, inhibition of reactive oxygen species/TGF cell Smad2/3 signaling axis, and superoxide dismutase 2 dependent mitochondrial regulation [10, 11]. In addition, Wang et al. reported that high serum irisin levels are associated with increased rates of adverse cardiovascular outcomes [12]. However, no predictive value analysis was performed in this study, and serum irisin concentrations were measured 28 days after PCI [12]. This work investigated the relationship between serum irisin level at admission and the severity of AMI and examined its predictive value for the occurrence of MACE within one year after PCI.

## Materials and methods

### Study design

**Figure 1.**
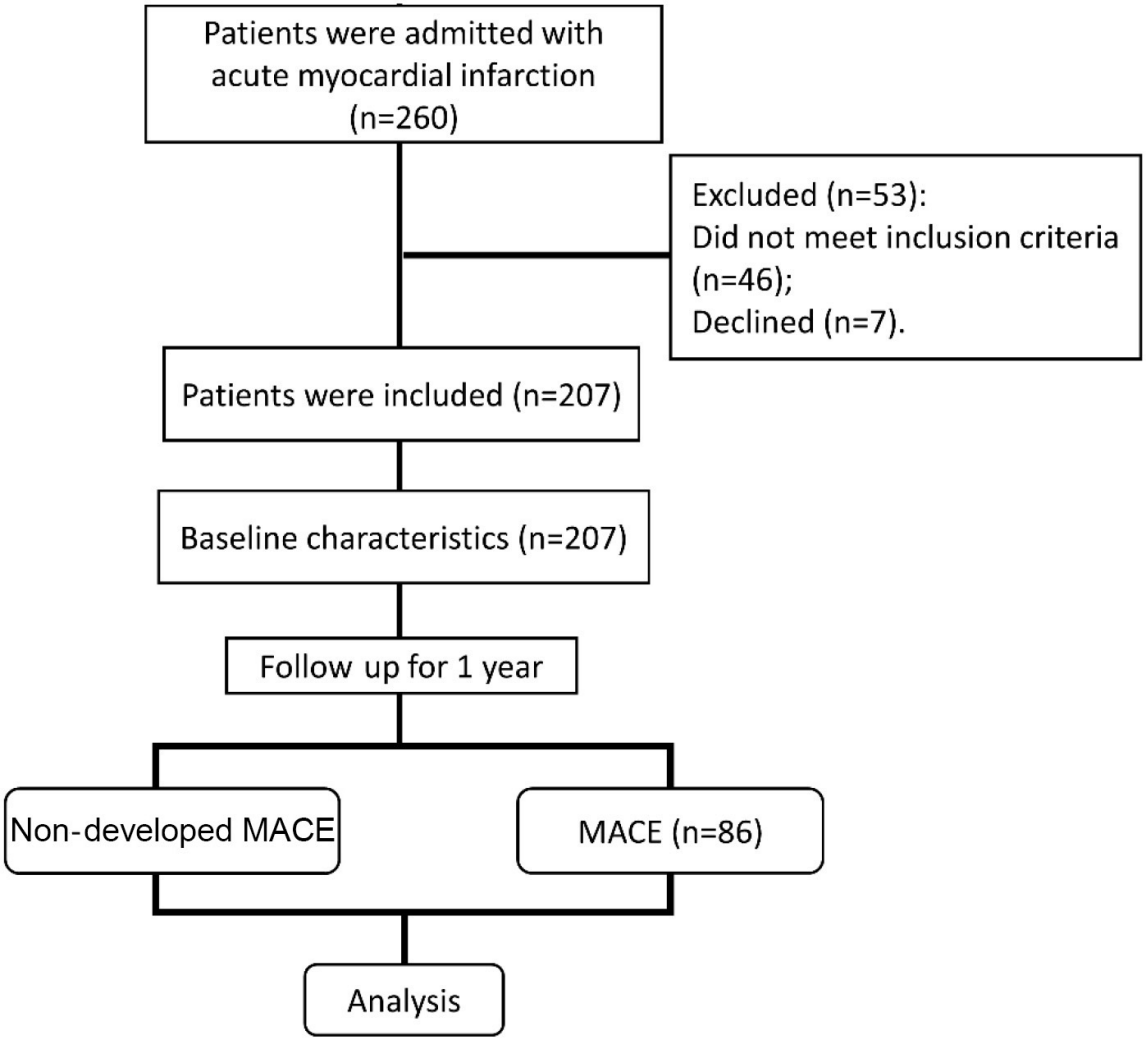
**Study design.** MACE: Major adverse cardiovascular events.

The flowchart of the study is shown in Figure 1. In this study, 260 patients with AMI who underwent PCI in the Cardiology Department of our hospital from September 2020 to November 2021 were initially selected as the research subjects. After 53 patients were excluded, 207 patients were finally included as the study subjects and the baseline characteristics were collected. After one year of follow-up, 86 patients developed MACE (MACE group) and 121 patients did not develop MACE (NMACE group).

### Patients

As a retrospective study, this study selected patients with AMI who underwent PCI in our hospital as the research subjects. A total of 207 patients were included, and their baseline data are shown in Table 1.

**Table 1:** Baseline characteristics of patients with and without postoperative major adverse cardiovascular events onset at one-year following up acute myocardial infarction

	**Non-developed MACE (*n* ═ 121)**	**MACE (*n* ═ 86)**	***p* value**
Age (years)	60.84 ± 9.55	65.52 ± 10.58	0.001
Body mass index (kg/m^2^)	23.42 ± 3.36	23.93 ± 3.45	0.257
*Sex (n, %)*			
Male	58 (47.9)	45 (52.3)	0.574
Female	63 (52.1)	41 (47.7)	
*AMI class (n, %)*			
STEMI	38 (31.4)	36 (41.9)	0.142
NSTEMI	83 (68.6)	50 (58.1)	
*Complication (n, %)*			
Diabetes mellitus	24 (19.8)	21 (24.4)	0.495
Hypertension	35 (28.9)	34 (39.5)	0.135
Hyperlipidemia	29 (23.9)	27 (31.4)	0.268
*Killip class at admission (n, %)*			
1	82 (67.8)	39 (45.3)	0.005
2	18 (14.9)	14 (16.3)	
3	12 (9.9)	17 (19.8)	
4	9 (7.4)	16 (18.6)	
Heart rate (b.p.m.)	76.66 ± 13.68	80.25 ± 14.63	0.088
SBP (mmHg)	125.7 ± 18.9	132.9 ± 21.7	0.209
DBP (mmHg)	82.4 ± 16.9	88.6 ± 18.4	0.153
LVEF (%)	55.71 ± 7.43	50.63 ± 7.22	<0.001
HDL-C (mmol/L)	1.39 ± 0.62	1.32 ± 0.57	0.272
LDL-C (mmol/L)	2.71 ± 0.92	3.11 ± 0.95	0.194
TC (mmol/L)	3.83 ± 1.09	4.11 ± 1.15	0.117
TG (mmol/L)	1.39 ± 0.54	1.58 ± 0.61	0.301
cTnI (ng/mL)	5.26 ± 2.63	7.07 ± 3.89	<0.001
CK-MB (ng/mL)	121.89 ± 43.34	187.52 ± 49.53	<0.001
Serum irisin (ng/mL)	78.93 ± 35.53	49.35 ± 28.11	<0.001

The data presented are mean ± SD or *n* (percentage). The comparisons of data between the MACE and non-developed MACE groups were done by Mann–Whitney test or Fisher’s exact test or chi-square test. MACE: Major adverse cardiovascular events; STEMI: ST-elevation myocardial infarction; NSTEMI: Non-ST-elevation myocardial infarction; SBP: Systolic blood pressure; DBP: Diastolic blood pressure; LVEF: Left ventricular ejection fraction; HDL-C: High density lipoprotein cholesterol; LDL-C: Low density lipoprotein cholesterol; TC: Total cholesterol; TG: Triglyceride; cTnI: Cardiac troponin I; CK-MB: Creatine kinase-muscle/brain; AMI: Acute myocardial infarction.

The diagnostic criteria for AMI were based on the “2015 China Emergency Acute Coronary Syndrome Clinical Practice Guidelines Diagnostic Criteria”, including the diagnosis of ST-segment elevation myocardial infarction (STEMI) and the diagnosis of non-ST-segment elevation myocardial infarction (NSTEMI).

Diagnostic criteria for STEMI were: severe chest pain lasting > 30 min; ST segment arched dorsal elevation on ECG; positive cardiac troponin T or I; hybrid creatine kinase isoenzyme level > 2 times the upper limit of the reference value.

Diagnostic criteria for NSTEMI were: persistent chest pain; transient or new ST segment depression on electrocardiogram, or T wave inversion and flattening; cardiac troponin T or I positive; hybrid creatine kinase isoenzyme level > 2 times the reference upper limit of value.

Inclusion criteria were: age ≥ 18 years; time from AMI to admission to hospital < 12 h; agreed to participate in this study; patients successfully achieved vascular recanalization by PCI.

Exclusion criteria were: patients with old myocardial infarction; patients with chronic heart failure; patients with pulmonary hypertension, pulmonary heart disease or severe liver and renal insufficiency and craniocerebral diseases; patients with congenital heart disease, cardiac valve disease and pericarditis; patients with malignant tumors; patients with a previous history of myocardial infarction or heart failure; estimated life expectancy <1 year.

The endpoint MACE mainly included readmission for unstable angina, new acute heart failure, recurrent AMI, cardiogenic shock/death, repeated revascularization, and malignant (severe) arrhythmia.

### Emergency percutaneous coronary intervention

PCI was performed in patients with confirmed AMI by a cardiologist within the emergency PCI time window. The patient lied flat on the operating table in the digital subtraction angiography room. Routine disinfection and puncture of the radial artery were performed, a 5F sheath was placed, and 6000 units of heparin was injected. Judkin left and right coronary angiography catheters were sequentially inserted through the sheath.

### Serum irisin level detection

Blood was collected from the median cubital vein before PCI. After standing at room temperature for 2 h, blood samples were centrifuged at 2000 r/min for 2 min, and serum was separated and stored in a –80 ^∘^C refrigerator. The level of irisin in serum was detected by irisin ELISA kit (Elabscience, Houston, TX, USA).

### Ethical statement

This study was approved by the Ethics Committee of the First Hospital of HanDan (2020/k35). The study was performed in strict accordance with the Declaration of Helsinki, Ethical Principles for Medical Research Involving Human Subjects. The patients or their families signed the informed consent.

### Statistical analysis

Data were presented as mean ± SD or *n* (percentage). The comparisons of data were done by Mann–Whitney test or Fisher’s exact test or chi-square test. The predictive value of irisin was performed by receiver operating characteristic (ROC) curve. Spearman correlation analysis was used to evaluate the relationship between serum irisin levels at admission and baseline characteristics. GraphPad was used for ROC analysis, with NMACE as the control data group and MACE as the patient data group. The Wilson/Brown method was used for ROC analysis. The serum irisin levels at admission at the maximum value of the Youden index were used to predict MACE occurring one year after AMI.

## Results

### Baseline characteristics of the study subjects

Baseline characteristics of patients at admission were compared between the MACE group and the NMACE group (Table 1). No significant differences were observed in body mass index, gender, AMI class, complications, heart rate, systolic blood pressure, diastolic blood pressure, high density lipoprotein cholesterol, low density lipoprotein cholesterol, total cholesterol, and triglyceride between the two groups. The mean ages of the MACE group and the NMACE group were 60.84 ± 9.55 and 65.52 ± 10.58 years, respectively, showing a significant difference (*p* ═ 0.001). In addition, the MACE group had a higher proportion of Killip class 2–4 at admission (*p* ═ 0.005) and lower left ventricular ejection fraction (LVEF, *p* < 0.001). Compared with the NMACE group, the MACE group had higher levels of commonly used diagnostic markers for AMI, including cardiac troponin I (cTnI, *p* < 0.001) and creatine kinase-muscle/brain (CK-MB, *p* < 0.001). Among the 207 patients included in the study, 74 were STEMI and 133 were NSTEMI. The incidence of postoperative MACE in patients with two different subtypes of concentric myocardial infarction was 38 and 36, respectively, with no significant difference (Figure S1). Notably, the serum irisin levels of the MACE group and the NMACE group were 78.93 ± 35.53 ng/mL and 49.35 ± 28.11 ng/mL, respectively, showing a significant difference (*p* < 0.001). Our results suggest that low serum irisin levels may be associated with the occurrence of MACE.

### Predictive value of serum irisin level at admission for patients with MACE

**Figure 2.**
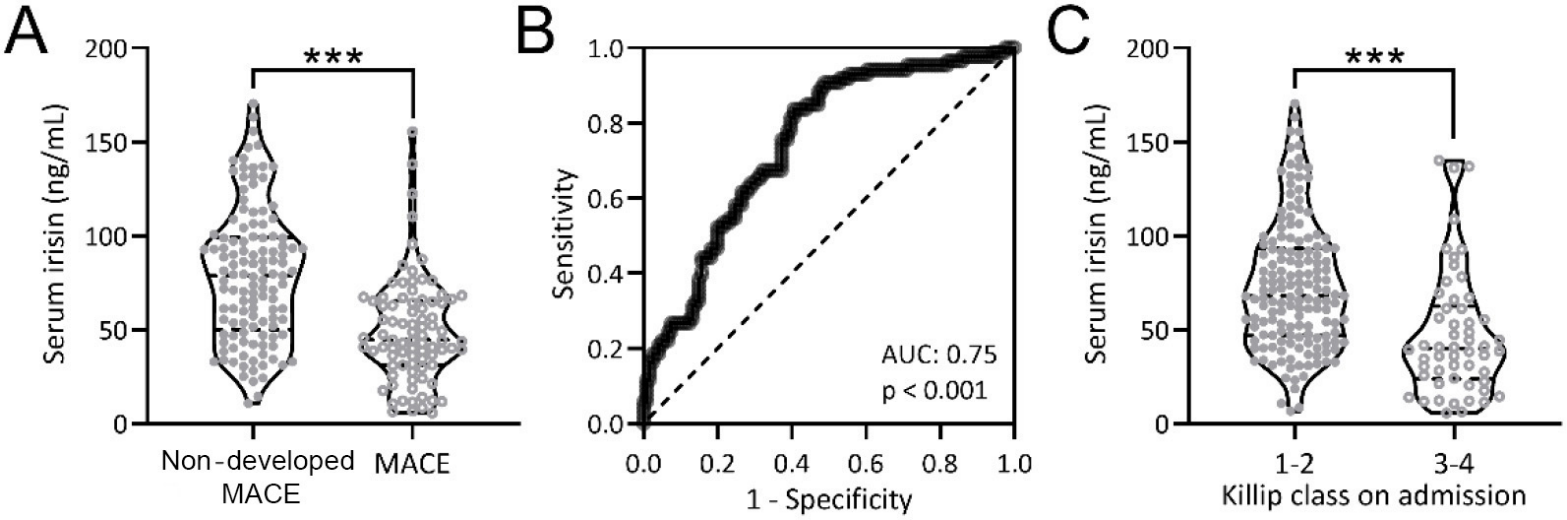
(A) Comparisons of serum irisin levels at admission between AMI patients with (*n* ═ 86) and without (*n* ═ 121) MACE onset at one-year follow-up; ***p < 0.001. Mann–Whitney test; (B) ROC analysis of serum irisin levels at admission for prediction of MACE onset at one-year following up in AMI patients; (C) Serum irisin levels at admission compared according to the Killip class. *n* ═ 152 for Killip class 1–2, *n* ═ 54 for Killip class 3–4; MACE: Major cardiovascular events; AUC: Area under curve; ROC: Receiver operating characteristic; AMI: Acute myocardial infarction.

Subsequently, we compared the serum irisin concentration at admission in patients who developed postoperative MACE with those who did not develop postoperative MACE. It could be seen that the serum irisin concentration in patients with MACE was significantly lower than that in the NMACE group (Figure 2A; *p* < 0.001). The predictive value of serum irisin level at admission for patients with MACE within one year was analyzed by ROC, and the area under curve was 0.75, which was significant (*p* < 0.001). The specific cutoff value was 69.07 ng/mL, the sensitivity was 83.72%, and the specificity was 59.50% (Figure 2B). AMI patients were divided into class 1–2 and 3–4 according to the Killip classification. Our results indicated that patients with Killip class 3–4 had lower admission serum irisin levels (Figure 2C; *p* < 0.001), suggesting that low serum irisin concentration is negatively correlated with the severity of AMI.

### Correlation between postoperative MACE onset and serum irisin level at admission

We divided AMI patients into groups with serum irisin levels less than 69.07 ng/mL (*n* ═ 121) and greater than 69.07 ng/mL (*n* ═ 86) at admission according to the cutoff value in the ROC analysis. The results showed that the incidence of total MACE and various MACE events (including malignant arrhythmia, heart failure, recurrent myocardial infarction, recurrent angina requiring revascularization, and cardiac mortality) were significantly lower in the group with serum irisin level greater than 69.07 ng/mL (Table 2; *p* < 0.001).

**Table 2:** Comparisons of postoperative major adverse cardiovascular events onset at one-year following up acute myocardial infarction according to the cut off of serum irisin

	**Cell serum irisin < 69.07 ng/mL (*n* ═ 121)**	**Cell serum irisin > 69.07 ng/mL (*n* ═ 86)**	***p* value**
Without MACE (*n*, %)	49 (40.5)	72 (83.7)	<0.001
Malignant arrhythmia (*n*, %)	11 (9.1)	2 (2.3)	
Heart failure (*n*, %)	29 (23.9)	5 (5.8)	
Recurrent myocardial infarction (*n*, %)	10 (8.3)	3 (3.5)	
Recurrent angina requiring revascularization (*n*, %)	14 (11.6)	3 (3.5)	
Cardiac mortality (*n*, %)	8 (6.6)	1 (1.2)	
Total MACE (*n*, %)	72 (59.5)	14 (16.3)	<0.001

MACE: Major adverse cardiovascular events.

### Correlation between severity of AMI and serum irisin level at admission

**Figure 3.**
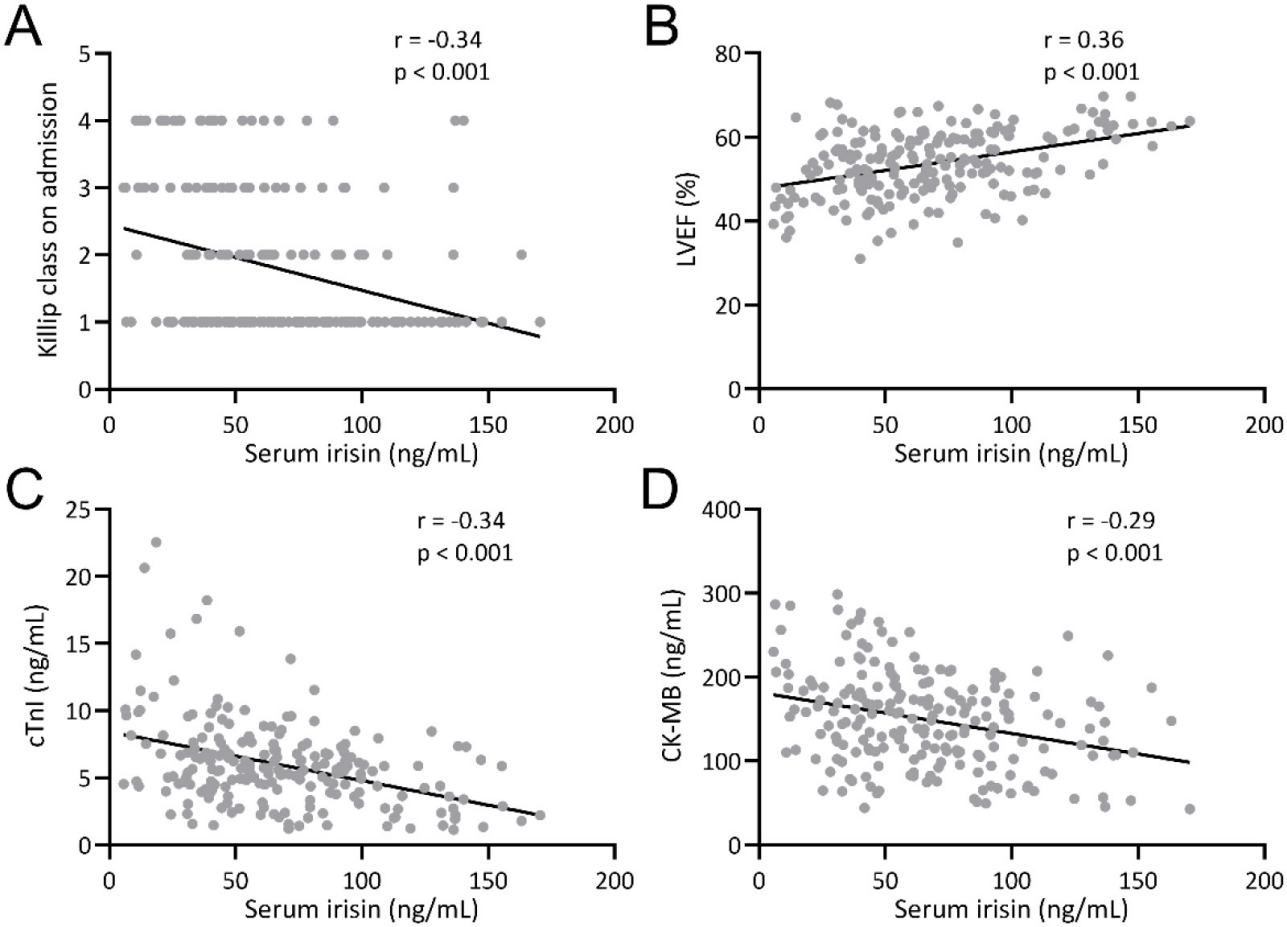
**Correlation of irisin levels with Killip class (A), left ventricular ejection fraction (B), serum cardiac troponin I (C), and creatine kinase muscle/brain (D).** LVEF: Left ventricular ejection fraction; cTnI: Cardiac troponin I; CK-MB: Creatine kinase-muscle/brain.

Subsequently, we analyzed the association between serum irisin levels at admission and AMI severity in all AMI patients (*n* ═ 207). Spearman correlation analysis showed that serum irisin levels at admission were significantly negatively correlated with Killip class (Figure 3A; *r* ═ –0.34, *p* < 0.001), and significantly positively correlated with LVEF (Figure 3B; *r* ═ 0.36, *p* < 0.001). Examining two commonly used diagnostic markers for AMI, we found that serum irisin levels at admission were significantly negatively correlated with serum cTnI (Figure 3C; *r* ═ –0.34, *p* < 0.001) and CK-MB (Figure 3D; *r* ═ –0.29, *p* < 0.001).

## Discussion

AMI caused by coronary atherosclerotic heart disease has a very high fatality and disability rate [13, 14]. Although with the development of medical technology, more and more AMI patients have received timely PCI treatment, which has greatly reduced the fatality rate of AMI [15]. However, there is still a certain incidence of poor prognosis after AMI revascularization, which has become a difficult problem in the treatment of AMI by PCI [16]. PCI can effectively restore myocardial blood perfusion [17]. However, ischemic myocardial reperfusion can further aggravate myocardial injury, cause myocardial cell death, and enlarge infarct size [18, 19]. This myocardial ischemia-reperfusion injury leads to further deterioration of cardiac function and MACE in patients with AMI [20]. Therefore, identifying reliable biomarkers for the prognosis of AMI is useful for reducing myocardial infarct size, preventing left ventricular remodeling, and reducing the occurrence of MACE.

In this study, we innovatively reported the predictive value of irisin, a newly discovered myogenic cytokine, for the occurrence of MACE within one year after PCI. As a retrospective study, we followed patients undergoing PCI for one year and divided patients into groups with and without MACE. The differences in parameters at baseline (at admission) were compared between the two groups to investigate the predictive value of serum irisin levels for patients developing MACE within one year. The ROC analysis of serum irisin level at admission for patients and MACE within one year suggests that the area under the curve was 0.75, which was significant (*p* < 0.001). Interestingly, our study also found that irisin levels at admission in AMI patients were also associated with the severity of AMI.

Irisin is mainly released from skeletal muscle and is also expressed in cardiac muscle, liver, lung, neurons, and other tissues [8]. Emerging research has shown that irisin could promote glucose metabolism, lipid metabolism, and weight loss, which is mainly related to various metabolic diseases [21]. In addition, irisin also plays an important role in cardiovascular disease, elderly Alzheimer’s disease, and tumors [22]. MACE after PCI in patients with AMI is mainly related to intracellular calcium overload, oxidative stress, inflammation, energy metabolism disorders, and apoptosis [20]. Irisin can significantly increase the rate pressure product and reduce left ventricular end-diastolic pressure to improve ventricular function [23]. Irisin has a certain protective effect on ischemia-reperfusion injury of brain, lung, liver, and other tissues and organs [24]. It has been reported that irisin inhibits cardiomyocyte apoptosis by promoting the degradation of histone deacetylase 4. Irisin can interact with superoxide dismutase-2 (SOD-2) and increase the activity of SOD-2 [25]. Irisin also restores the localization of SOD-2 in mitochondria, thereby inhibiting oxidative stress and protecting mitochondrial function in cardiomyocytes [26]. In conclusion, irisin can protect the heart of patients after PCI by inhibiting cardiomyocyte apoptosis, improving myocardial mitochondrial function, and inhibiting local inflammatory response caused by AMI. Consistent with previous studies, we found a statistically significant negative correlation between serum irisin levels at admission and AMI severity in AMI patients in this study, suggesting a potential cardioprotective role for irisin. The predictive value of serum irisin level at admission for patients with MACE within one year was analyzed by ROC, and the area under the curve was 0.75, which was significant.

The functions and direct effects of irisin have been investigated meticulously in recent years, but the receptors of irisin and the downstream signal transduction pathways involved are still unclear. Some studies suggest that irisin regulates the browning of white adipocytes [7] and the proliferation of osteoblasts [27] by activating the kinase function of the mitogen-activated kinase. In addition, irisin inhibits hepatic cholesterol synthesis through 5’-phosphate-activated protein kinase [28]. Irisin enhances the phosphorylation of AKT and promotes cardiomyocyte proliferation and mitochondrial metabolism in cardiomyocytes [29]. A recent study has shown that irisin could induce angiogenesis in human umbilical vein endothelial cells and zebrafish [30]. These previous findings indicate that irisin may play a long-lasting role in vascular remodeling, which might be the reason for MACE onset. The response of the myocardium to acute exercise produces more irisin than skeletal muscle and is associated with myocardial infarction. In addition to cardiac troponin and CK-MB, iridin provides new diagnostic information for AMI patients, and a gradual decrease in saliva/serum irisin within 48 h may be a useful biomarker [31]. In addition, a study reported that serum irisin levels were significantly increased in patients with coronary artery disease, and serum irisin levels were interrelated with prognosis in patients with coronary artery disease after PCI [32]. Another study suggested that circulating irisin levels could not predict the development of acute coronary syndromes in healthy individuals. An increase in irisin levels is associated with the development of MACE in patients with confirmed coronary artery disease after PCI [33].

In fact, there are still some deficiencies in our research. We only followed up MACE within one year after PCI in 207 patients. The comorbidities and medication conditions of different patients are likely to affect the occurrence and development of MACE. We plan to conduct a multicenter study in the future to expand the sample size and further group to exclude the influence of other factors.

## Conclusion

Our study revealed that patients with high serum irisin levels at admission had a lower probability of MACE after PCI. In addition, serum irisin levels at admission in AMI patients were significantly correlated with the Killip class, serum cTnI, and CK-MB levels and could be used as an effective biomarker to predict the onset of MACE in AMI patients after PCI.

## Supplemental Data

**Figure S1.**
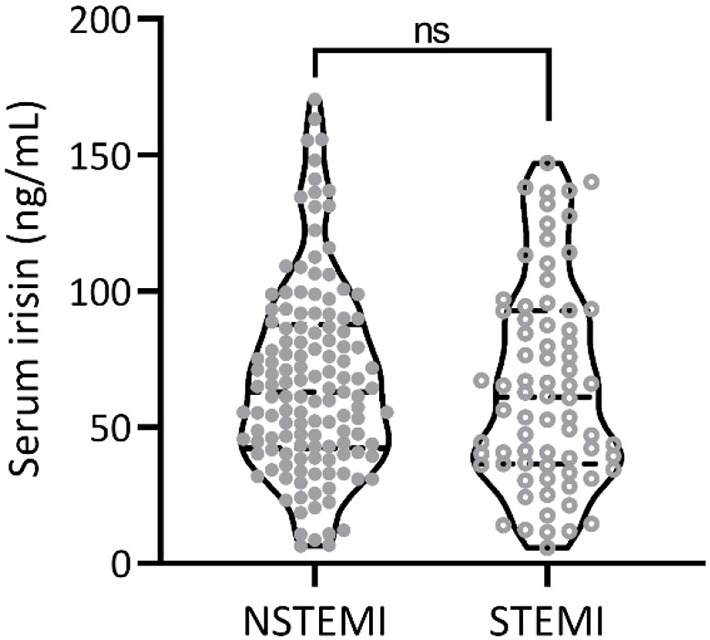
**Comparison of serum irisin levels at admission between ST-elevation myocardial infarction patients (STEMI) and non-ST-elevation myocardial infarction (NSTEMI) patients.** ns: No significance.
